# Exogenous lipoid pneumonia caused by repeated sesame oil pulling: a report of two cases

**DOI:** 10.1186/s12890-015-0134-8

**Published:** 2015-10-30

**Authors:** Muneyoshi Kuroyama, Hiroyuki Kagawa, Seigo Kitada, Ryoji Maekura, Masahide Mori, Hiroshi Hirano

**Affiliations:** Department of Respiratory Medicine, National Hospital Organization Toneyama National Hospital, Toneyama, 5-1-1, Toyonaka, Osaka 560-8552 Japan; Department of Respiratory Medicine, Allergy, and Rheumatic Diseases, Osaka University Graduate School of Medicine, Suita, Osaka, Japan; Department of Pathology, National Hospital Organization Toneyama National Hospital, Toyonaka, Osaka, Japan

**Keywords:** Lipoid pneumonia, Sesame oil pulling, Alveolar macrophages

## Abstract

**Background:**

Exogenous lipoid pneumonia is a rare disease caused by aspiration or inhalation of oily substances.

**Case presentation:**

A 66-year-old male with dry cough (Case 1) and a 38-year-old female with shortness of breath (Case 2) demonstrated ground-glass opacities on chest computed tomography and were diagnosed with lipoid pneumonia based on the confirmation of lipid-laden alveolar macrophages. Both patients habitually performed sesame oil pulling via nasal or mouth washing for several months prior to the diagnosis.

**Conclusion:**

Steroid therapy and bronchoalveolar lavage resulted in improvement in Case 1, and no intensive therapy was required for Case 2. Sesame oil pulling has been rarely been reported to cause lipoid pneumonia.

## Background

Lipoid pneumonia is an uncommon non-infectious inflammatory lung disease that is caused by the presence of lipids in the alveoli [1]. It is classified into two major groups, depending on whether the lipid/oil in the respiratory tract is from an exogenous or endogenous/idiopathic source [2]. Pathologically, lipoid pneumonia is a chronic foreign body reaction to fat. It is characterized by lipid-laden macrophages. Although there have been reports on exogenous lipoid pneumonia caused by various types of lipids and oils [3–8], to the best of our knowledge, only one report has indicated that oil pulling (specifically sesame oil pulling), was a cause of lipoid pneumonia [9]. We herein report two uncommon cases of lipoid pneumonia that occurred due to repeated sesame oil pulling.

## Case presentation

### Case 1

**Fig. 1:**
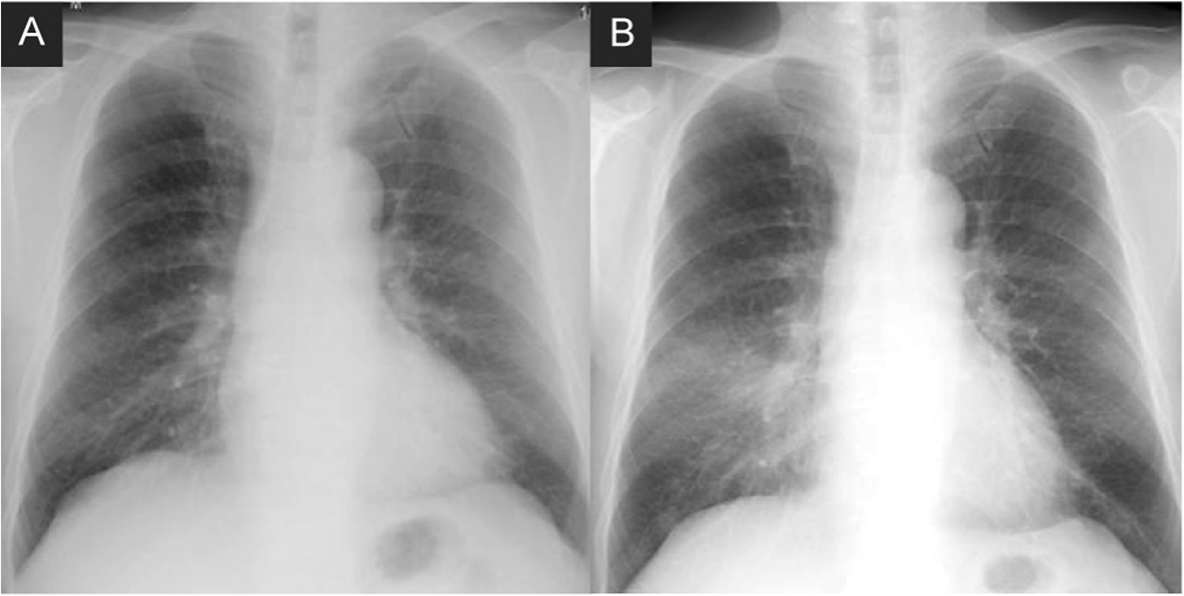
The chest roentgenogram of a 66-year-old male with lipoid pneumonia. No abnormal shadows were detected 6 months prior to admission (**a**). Infiltrative shadows were noted in the bilateral lower lung fields on admission (**b**)

A 66-year-old male who was a former smoker (67 pack-years) presented with a chief complaint of dry cough. He had no relevant medical history and was not taking any prescribed medications. He habitually performed sesame oil pulling via nasal washing every evening for 8 months and often aspirated the oil. Three months after the onset of symptoms, he was admitted to our hospital for a detailed examination of abnormal chest shadows (Fig. 1b), which had not been detected 6 months previously (Fig. 1a). The following clinical observations were recorded at admission: respiratory rate, 18 breaths/min; blood pressure, 132/84 mmHg; body temperature, 36.0 °C; and oxygen saturation, 97 %.

**Fig. 2:**
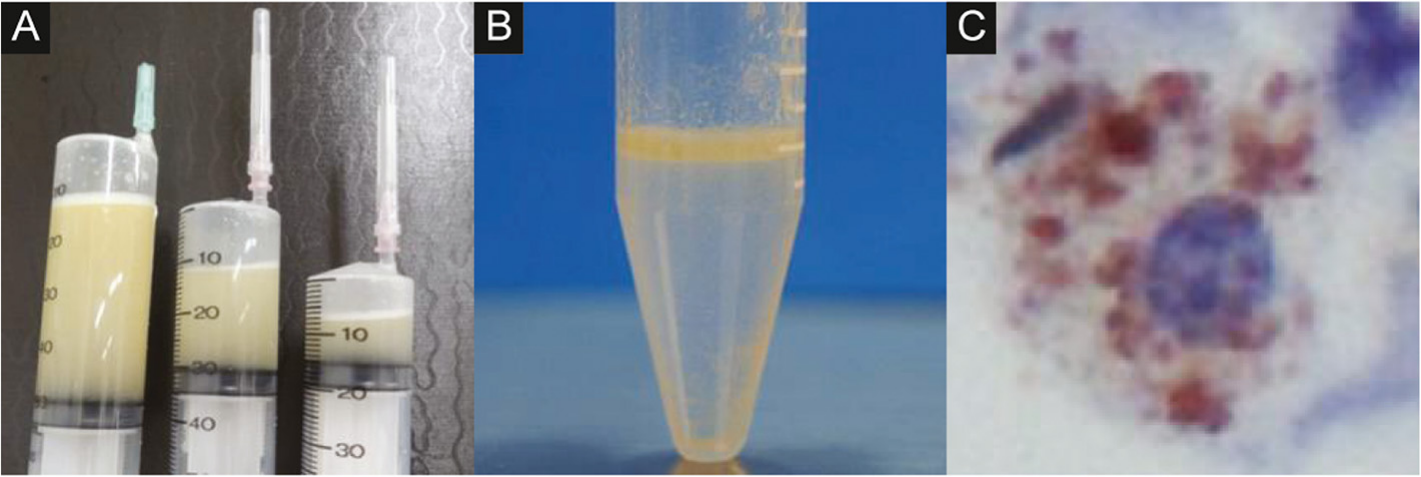
The bronchoalveolar fluid examination in a 66-year-old male with lipoid pneumonia. The bronchoalveolar lavage fluid was initially turbid white (**a**) and subsequently exhibited a bilayer appearance (**b**). Oil phagocytosis by alveolar macrophages was observed under a microscope with Sudan III staining (**c**)

A physical examination revealed no abnormal findings, with the exception of inspiratory fine crackles in the right lower lung field on auscultation. The patient had no fever and the results of routine laboratory tests (including complete blood count, erythrocyte sedimentation rate, C-reactive protein and blood biochemistry) were normal, with the exception of his lactate dehydrogenase (LDH) and Krebs Von Den Lungen-6 (KL-6) levels, which were 249 IU/mL and 615 IU/L, respectively.

**Fig. 3:**
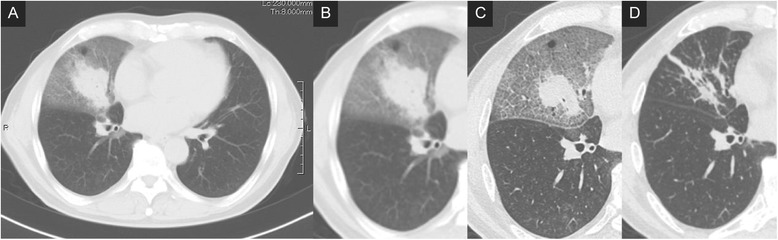
Serial chest computed tomography images of a 66-year-old male with lipoid pneumonia. Ground-glass opacities with interlobular septal thickening (crazy-paving pattern) and partial infiltration in the right middle and bilateral lower lobes were observed on admission (**a, b**). The infiltrative shadow showed slight improvement after steroid pulse therapy (**c**). Subsequent bronchoalveolar lavage resulted in significant improvement (**d**)

A chest roentgenogram and computed tomography (CT) revealed ground-glass opacity and interlobular septal thickening in the bilateral middle and lower lobes of the lungs that were particularly dominant in the right middle lobe (Figs. 1b and 3a).

Bronchoalveolar lavage fluid (BALF) obtained from the right B4 bronchus was initially turbid white (Fig. 2a) with a subsequent bilayer appearance (Fig. 2b). Cultures and stains of the BALF specimen were negative for infectious organisms. The total cell count of the BALF was 439,000/ml. The cells consisted of macrophages (33 %), lymphocytes (60 %), neutrophils (4 %), and of eosinophils (3 %). The upper layer of the BALF was confirmed to have an oil component based on the microscopic detection of oil phagocytosis by alveolar macrophages that were stained with Sudan III (Fig. 2c). Thus, he was diagnosed with lipoid pneumonia.

The patient received steroid pulse therapy with methylprednisolone (1 g) for 3 days, followed by prednisolone (20 mg), resulting in a mild improvement (Fig. 3c). However, the infiltrative lung shadow showed re-progression 2 months after the dose of predonisolone was reduced. Bronchoalveolar lavage of the right middle lobe (20 aliquots of 50 mL saline) was performed under general anesthesia 4 months after the diagnosis. The lung infiltration regressed after this treatment (Fig. 3d), and oral prednisolone therapy was continued and gradually tapered for 8 months. There were no signs of recurrence.

### Case 2

A 38-year-old female who was a non-smoker with no history of smoking had recently become short of breath. She had no relevant medical history, and was not receiving any prescribed medications. She habitually performed sesame oil pulling via mouth washing every morning for 6 months and would sometimes aspirate the oil. Five months after the onset of symptoms, she was admitted to our hospital for a detailed examination of abnormal chest shadows that were detected during a medical check-up. The following clinical observations were recorded at admission: respiratory rate, 16 breaths/min; blood pressure, 130/60 mmHg; body temperature, 36.3 °C; and oxygen saturation, 98 %.

**Fig. 4:**
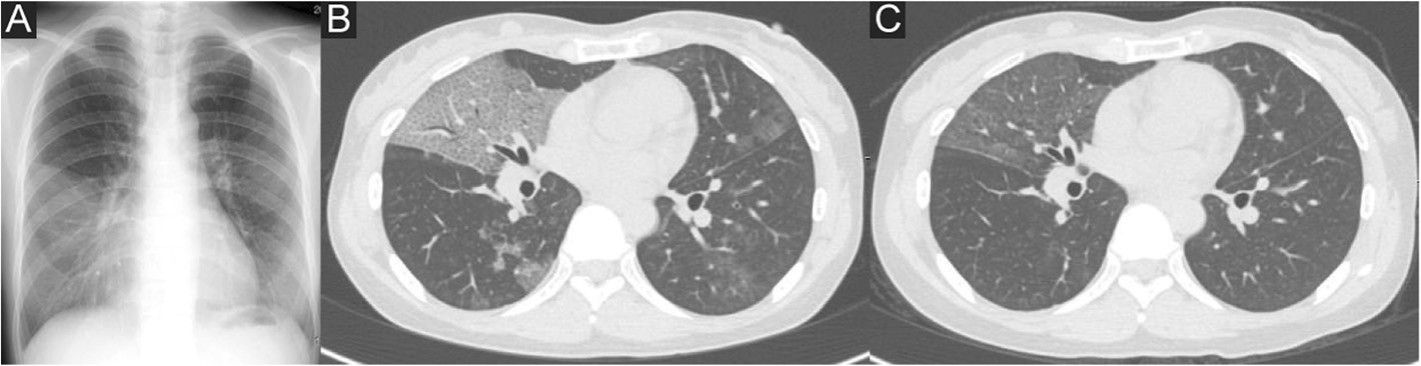
A chest roentgenogram and computed tomography images of a 38-year-old female with lipoid pneumonia. Infiltrative shadows were noted in the bilateral lower lung fields on admission (**a**). Chest CT revealed ground-glass opacities with interlobular septal thickening (crazy-paving pattern) and partial infiltration in the bilateral lower lung fields (**b**). The shadows improved 3 months later (**c**)

The patient had no fever and the results of routine laboratory tests (including complete blood count, erythrocyte sedimentation rate, C-reactive protein and blood biochemistry) were all normal; however, a chest roentgenogram and CT revealed ground-glass opacities in the bilateral middle and lower lobes that were particularly dominant in the middle and lingular lobes (Fig. 4a, b). Biochemistry results and peripheral blood examination results were normal, with the exception of the patient’s LDH level, which was 250 IU/ mL.

**Fig. 5:**
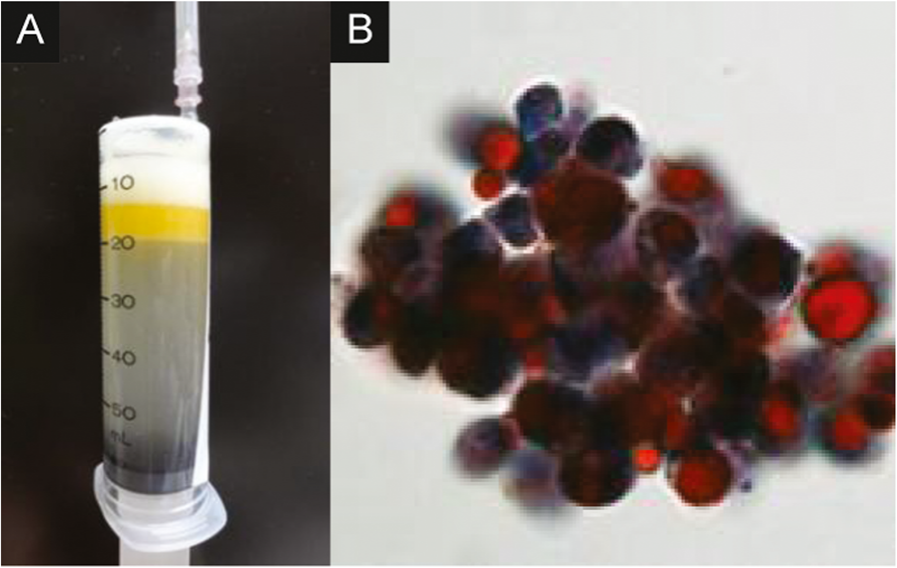
The bronchoalveolar fluid examination in a 38-year-old female with lipoid pneumonia. The bronchoalveolar lavage fluid had a bilayer appearance (**a**). Oil phagocytosis by alveolar macrophages was observed under a microscope with Oil Red staining (**b**)

The patient underwent bronchofiberscopy; the BALF from the right B4 bronchus demonstrated findings that were similar to Case 1 (Fig. 5a). The total cell count of the BALF was 156,000/ml. The cells consisted of macrophages (82 %), lymphocytes (8.7 %), neutrophils (9.3 %), and eosinophils (0 %). The upper layer of the BALF was confirmed to have an oil component based on the microscopic detection of oil phagocytosis by alveolar macrophages, which were stained with Sudan III (Fig. 5b) . She was therefore diagnosed with lipoid pneumonia due to sesame oil pulling. She was closely followed up. She discontinued oil pulling and showed gradual improvement 3 months later without intensive treatment (Fig. 4c).

## Discussion

Lipoid pneumonia is a rare type of pneumonia that was first reported by Laughlen in 1925 [10]. Thereafter, only sporadic cases have been reported. The disease is characterized by the presence of lipid-laden macrophages in the alveoli and is broadly divided into either endogenous or exogenous etiologies. Endogenous lipoid pneumonia may be primary or secondary to obstructive pneumonia and exhibits a chronic pattern in many cases. Moreover, it may be caused by the secretion of cholesterol and/or its ester derivatives from inflammatory lesions. In contrast, exogenous lipoid pneumonia results from the inhalation or aspiration of oil components. Exogenous cases may manifest with either an acute pattern, due to the inhalation of large amounts of oil components within a short period of time; or a chronic pattern, resulting from the deposition of small amounts of oil over long periods of time [11–13].

Exogenous lipoid pneumonia has been reported to be caused by various types of lipids and oils. There have been many reports on liquid paraffin [3] and laxatives [4] as causative agents. Other causes include being a professional fire eater [5], insecticide inhalation [6], pulmonary aspiration of kerosene [7], and the use of oil-based nose drops [8].

Oil pulling has also been reported to induce lipoid pneumonia following habitual mouth washing with oil [9], as was observed in this report in the patient of Case 2. The patient of Case 1 also used sesame oil for nasal washing. Both patients often aspirated sesame oil, indicating that sesame oil pulling may have induced lipoid pneumonia. To the best of our knowledge, there has been only one report to indicate oil pulling (specifically sesame oil pulling) was a cause of lipoid pneumonia.

Oil pulling has been used extensively as a traditional Indian folk remedy for many years to prevent decay, oral malodor, bleeding gums, dryness of throat, cracked lips and for strengthening the teeth, gums and the jaw. Oil pulling is a procedure that involves swishing oil in the mouth for oral and systemic health benefits. It is mentioned in the Ayurvedic text, *Charaka Samhita*, where it is called “kavala” or “gandusha,” and is claimed to cure about 30 systemic diseases ranging from headache, migraine to diabetes and asthma [14, 15]. Oil pulling therapy can be performed using oils like sunflower oil or sesame oil. This method appears to have gained popularity in Japan after being introduced on a popular television show and has been popularized on the Internet. Sesame oil, through both the oral and nasal washing routes, appears to be the most commonly used oil. However, the number of individuals that engage in oil pulling in Japan is unknown.

The symptoms that are commonly observed in cases of exogenous lipoid pneumonia include fever, weight loss, cough, dyspnea, chest pain, and hemoptysis. However, the disease is difficult to diagnose because approximately 40 % of lipoid pneumonia patients have only mild symptoms or no symptoms at all, thus it is often found incidentally [2, 16]. In this study, both patients had mild symptoms of dry cough and dyspnea.

In the present cases, chest imaging revealed various characteristic features, including airspace consolidation, ground-glass attenuation, and mass shadows, which were accompanied by interlobular septal thickening (crazy-paving pattern) [17]. Most of the lesions were unilaterally dominant in the lower lobe or the right middle lobe, which are locations that are dependent on the patient’s sleeping position [18]. The patient in Case 1 often engaged in nasal washing with sesame oil immediately before sleeping and tended to sleep on the right side, which supports the above assumption.

Acute pattern cases sometimes involve the development of severe pneumonia and may be fatal [12], whereas many chronic pattern cases of exogenous lipoid pneumonia are characterized by minimal symptoms. The type and volume of lipid inhalation or aspiration are related to the exogenous onset and severity of lipoid pneumonia [19].

Few reviews have presented information regarding the systematic treatment of lipoid pneumonia. However, at a minimum, the source of exposure to the causative lipid or oil must be removed. In mild cases, such as Case 2 of the present report, spontaneous remission is often achieved after the discontinuation of the causative stimuli and conservative management [18]. In severe cases, oxygen inhalation or mechanical ventilation is required. Steroid therapy may be effective for treating cases in which lipoid pneumonia is associated with macrophage activation as a consequence of chronic inhalation of the lipid [20]; however, not all cases respond to this treatment [21]. Repeated exposure to oil components and lung inflammation may account for irreversible lung damage in such cases. In fact, prednisolone induced only a slight improvement in Case 1.

The mechanical removal of the oil components by alveolar lavage, similar to the method applied for pulmonary alveolar proteinosis, has also been reported to improve severe cases of lipoid pneumonia [22, 23]. In Case 1, bronchoalveolar lavage resulted in improvement.

We reported two rare cases of lipoid pneumonia that were caused by repeated sesame oil pulling. Sesame oil pulling should therefore be considered as a possible cause of lipoid pneumonia in patients who live in regions where this custom is popular.

## Conclusion

Sesame oil pulling should be considered as a possible cause of lipoid pneumonia in patients who live in regions where the custom is popular.

## Consent

Written informed consent was obtained from the patients for the publication of this case report and any accompanying images. A copy of the written consent is available for review by the editor of this journal.

## References

[1] Himsworth CG, Malek S, Saville K, Allen AL (2008). Pathologists’ corner: endogenous lipid pneumonia and what lies beneath. Can Vet J.

[2] Hadda V (2010). Lipoid pneumonia: an overview. Expert Rev Respir Med.

[3] Ohwada A, Yoshioka Y, Shimanuki Y, Mitani K, Kumasaka T, Dambara T (2002). Exogenous lipoid pneumonia following ingestion of liquid paraffin. Intern Med.

[4] de Albuquerque Filho AP (2006). Exogenous lipoid pneumonia: importance of clinical history to the diagnosis. J Bras Pneumol.

[5] Dell’ Omo M, Murgia N, Chiodi M, Giovenali P, Cecati A, Gambelunghe A (2010). Acute pneumonia in a fire-eater. Int J Immunopathol Pharmacol.

[6] Yokohori N, Homma S, Tanaka S, Kawabata M, Kishi K, Tsuboi E, et al. Exogenous lipoid pneumonia induced by inhalation of insecticide. Nihon Kokyuki Gakkai Zasshi*.* 10481467

[7] Gotanda H, Kameyama Y, Yamaguchi Y, Ishii M, Hanaoka Y, Yamamoto H (2013). Acute exogenous lipoid pneumonia caused by accidental kerosene ingestion in an elderly patient with dementia: a case report. Geriatr Gerontol Int.

[8] Spatafora M, Bellia V, Ferrara G, Genova G (1987). Diagnosis of a case of lipoid pneumonia by bronchoalveolar lavage. Respiration.

[9] Kim JY, Jung JW, Choi JC, Shin JW, Park IW, Choi BW (2014). Recurrent lipoid pneumonia associated with oil pulling. Int J Tuberc Lung Dis.

[10] Laughlen GF (1925). Studies on pneumonia following nasopharyngeal injections of oil. Am J Pathol.

[11] Paraskevaides EC (1990). Fatal lipid pneumonia and liquid paraffin. Br J Clin Pract.

[12] Soloaga ED, Beltramo MN, Veltri MA, Ubaldini JE, Chertcoff FJ (2000). Acute respiratory failure due to lipoid pneumonia. Medicina (B Aires).

[13] Hochart A, Thumerelle C, Petyt L, Mordacq C, Deschildre A. Chronic lipoid pneumonia in a 9-year-old child revealed by recurrent chest pain. Case Rep Pediatr. 2015;402926. doi: 10.1155/2015/402926. Epub 2015 May 21. 10.1155/2015/402926 PMC4454704 26078902

[14] Bethesda M. A Closer Look at Ayurvedic Medicine. Focus on Complementary and Alternative Medicine. Maryland: National Center for Complementary and Alternative Medicine, US National Institutes of Health. 2006;XII(4)

[15] Hebbar A, Keluskar V, Shetti A (2010). Oil pulling – unraveling the path to mystic cure. J Int Oral Health.

[16] Gondouin A, Manzoni P, Ranfaing E, Brun J, Cadranel J, Sadoun D (1996). Exogenous lipid pneumonia: a retrospective multicenter study of 44 cases in France. Eur Respir J.

[17] Franquet T, Gimenez A, Bordes R, Rodriguez-Arias JM, Castella J (1998). The crazy-paving pattern in exogenous lipoid pneumonia: CT-pathologic correlation. AJR Am J Roentgenol.

[18] Spickard A, Hirschmann JV (1994). Exogenous lipoid pneumonia. Arch Intern Med.

[19] Gentina T, Tillie-Leblond I, Birolleau S, Saidi F, Saelens T, Boudoux L (2001). FireEater’s lung: seventeen cases and a review of the literature. Medicine (Baltimore).

[20] Chin NK1, Hui KP, Sinniah R, Chan TB (1994). Idiopathic lipoid pneumonia in an adult treated with prednisolone. Chest.

[21] Ayvazian LF, Steward DS, Merkel CG, Frederick WW (1967). Diffuse lipoid pneumonitis successfully treated with prednisone. Am J Med.

[22] Chang HY, Chen CW, Chen CY, Hsuie TR, Chen CR, Lei WW (1993). Successful treatment of diffuse lipoid pneumonitis with whole lung lavage. Thorax.

[23] Nakashima S, Ishimatsu Y, Hara S, Kitaichi M, Kohno S (2015). Exogenous lipoid pneumonia successfully treated with bronchoscopic segmental lavage therapy. Respir Care.

